# CD98 Heavy Chain Is a Potent Positive Regulator of CD4^+^ T Cell Proliferation and Interferon-γ Production *In Vivo*

**DOI:** 10.1371/journal.pone.0139692

**Published:** 2015-10-07

**Authors:** Takeshi Kurihara, Hideki Arimochi, Zaied Ahmed Bhuyan, Chieko Ishifune, Hideki Tsumura, Morihiro Ito, Yasuhiko Ito, Akiko Kitamura, Yoichi Maekawa, Koji Yasutomo

**Affiliations:** 1 Department of Immunology and Parasitology, Graduate School of Medicine, Tokushima University, Tokushima, Japan; 2 Division of Laboratory Animal Resources, National Research Institute for Child Health and Development, Tokyo, Japan; 3 Department of Biomedical Sciences, College of Life and Health Sciences, Chubu University, Aichi, Japan; INRS - Institut Armand Frappier, CANADA

## Abstract

Upon their recognition of antigens presented by the MHC, T cell proliferation is vital for clonal expansion and the acquisition of effector functions, which are essential for mounting adaptive immune responses. The CD98 heavy chain (CD98hc, *Slc3a2*) plays a crucial role in the proliferation of both CD4^+^ and CD8^+^ T cells, although it is unclear if CD98hc directly regulates the T cell effector functions that are not linked with T cell proliferation *in vivo*. Here, we demonstrate that CD98hc is required for both CD4^+^ T cell proliferation and Th1 functional differentiation. T cell-specific deletion of CD98hc did not affect T cell development in the thymus. CD98hc-deficient CD4^+^ T cells proliferated *in vivo* more slowly as compared with control T cells. C57BL/6 mice lacking CD98hc in their CD4^+^ T cells could not control *Leishmania major* infections due to lowered IFN-γ production, even with massive CD4^+^ T cell proliferation. CD98hc-deficient CD4^+^ T cells exhibited lower IFN-γ production compared with wild-type T cells, even when comparing IFN-γ expression in cells that underwent the same number of cell divisions. Therefore, these data indicate that CD98hc is required for CD4^+^ T cell expansion and functional Th1 differentiation *in vivo*, and suggest that CD98hc might be a good target for treating Th1-mediated immune disorders.

## Introduction

T cells express T cell receptors (TCR) that recognize antigens presented to them by the MHC and differentiate into various effector cells, which are essential for mounting defenses against pathogens [1] [2] [3]. However, excessive T cell responses contribute to various diseases, including autoimmune diseases [4] [5]. T cell proliferation and functional differentiation are regulated by signaling through the TCR, cytokines, and co-receptor molecules [6] [3]. Networks that incorporate these stimuli tightly regulate the acquisition of either effector or suppressive functions of mature T cells, which subsequently control T cell-mediated adaptive immune responses.

CD98 is comprised of a heavy and a light chain. Its heavy chain (CD98hc) is involved in integrin signaling and cell fusion, and its light chain controls amino-acid transport [7] [8] [9]. The *Slc3a2* gene encodes for CD98hc, and *Slc3a2* null mice exhibit embryonic lethality [10]. It has been shown that CD98hc controls T cell activation [11] and a recent report in which mice had *Slc3a2* deleted only in their T cells showed that CD98hc was important for T cell proliferation, but was not essential for T cell effector functions [12]. We previously reported that an anti-CD98hc mAb that could inhibit T cell proliferation suppressed the development of type1 diabetes [13]. These results suggest that CD98hc is crucial for T cell-mediated adaptive immune responses. However, it remains unclear if CD98hc is required for the acquisition of effector functions by CD4^+^ and CD8^+^ T cells *in vivo*.

Here, we investigated if CD98hc was required for functional CD4^+^ T cell differentiation *in vivo* using *Slc3a2* floxed mice. We found that *Slc3a2* deficiency disturbed both T cell proliferation and T cell effector functions. We determined that T cell specific-*Slc3a2* deficient mice under a C57BL/6 background could not control *Leishmania major* infection due to reduced IFN-γ production, even though CD4^+^ T cells proliferated vigorously. We also evaluated the secretion of IFN-γ by CD4^+^ T cells among cells undergoing division, which revealed that IFN-γ secretion was reduced due to CD98hc deficiency within each divided cell. These data indicate that CD98hc controls both CD4^+^ T cell proliferation and Th1 differentiation, suggesting that CD98hc is important for Th1 immune responses.

## Material and Methods

### Mice

Six- to 8-wk-old C57BL/6 mice were purchased from Japan SLC (Hamamatsu). *Slc3a2* ^flox/flox^ mice crossed with *CD4-Cre* transgenic mice were generated [14]. Thy1.1 or CD45.1 C57BL/6 mice and OT-II TCR transgenic mice were purchased from The Jackson Laboratory and Taconic Farms, Inc, respectively. All mice were housed under specific pathogen-free conditions. The studies in this manuscript were approved by the Committee on the Ethics of Animal Experiments of Tokushima University and the care and use of animals complied with institutional guidelines.

### Antibodies and flow cytometry

Fluorochrome-conjugated anti-CD3, CD4, CD8, CD44, CD25, and CD62L mAbs were purchased from BioLegend (CA, USA). Anti-CD98hc antibody was described previously [13]. APC-conjugated AnnexinV was purchased from BD Biosciences (NJ, USA). To detect intracellular expression of IFN-γ by flow cytometry, cells were stimulated with PMA (0.04 μM) and ionomycin (1.3 μM) for 5 hours in the presence of monensin (2 mM). Then, cells were stained with a PB-conjugated anti-CD4 mAb and fixed with 4% paraformaldehyde. After washing, cells were stained with APC-conjugated anti-IFN-γ (BioLegend) in a buffer containing saponin. Fluorescent signals were acquired with a FACS CantoII (BD Biosciences) and Flow-Jo software (Tree Star, Inc) was used for analysis.

### Cell culture

Draining lymph nodes and spleens were harvested and pooled for each experimental group. Immune cells from these tissues were isolated using standard methods and suspended in culture medium. Cells (5 x 10^5^ cells/well) in triplicate cultures (0.2 ml each) were stimulated with either an anti-CD3 mAb (1 μg/ml), ConA (5 μg/ml), or OVA protein (50 μg/ml) in 96-well round-bottom plates. Culture medium was RPMI 1640 supplemented with 2-ME, glutamine, non-essential amino acids, sodium pyruvate, antibiotics, and 10% fetal bovine serum. For some experiments, the following combination was also added to cultures for Th1 conditions: IL–12 (10 ng/ml) and anti-IL–4 mAb (10 μg/ml; e-Bioscience). Cells were cultured for 72 h; they were pulsed with [^3^H]-thymidine (1.0 μCi/10 μl/well) during the last 6 h to determine T cell proliferation. For some additional experiments, cells were also stimulated with OVA (323–339) peptides (1 μM) for 72 hours.

### *Leishmania major* infection

*Leishmania major* (MHOM/SU/73/5ASKH) parasites were grown in Schneider’s insect medium. Mice were infected in their hind footpads with 5 x 10^6^ parasites each after sedation with tribromo-ethanol. We euthanized mice infected with *Leishmania major* when footpad swelling was greater than 3 mm. Euthanasia was performed by carbon dioxide inhalation. Popliteal lymph node cells that harbored parasites were grown in Schneider’s medium containing 20% fetal bovine serum at 25°C for 5 days. Then, total parasite numbers in lymph node cells were determined. For T cell stimulation experiments, CD4^+^ T cells from popliteal lymph nodes were purified with CD4 T cell isolation kits (Milteny Biotec, Bergisch Gladbach, Germany). The purified CD4^+^ T cells (5 x 10^5^) were stimulated with 30 Gy-irradiated spleen cells (5 x 10^5^) in 96-well plates for three days in the presence *Leishmania majo*r-derived antigens, which were prepared as described [15]. These culture supernatants were used for determinations of IFN-γ and IL–4 by ELISA. In some experiments, cells were pulsed with [^3^H]-thymidine (1.0 μCi/10 μl/well), and [^3^H]-thymidine incorporation during the final 6 hours of culture was determined.

### T cell transfer

Mouse total spleen cells were labeled with CFSE as previously described [16]. CFSE-labeled T cells (5 x 10^6^) were transferred into irradiated CD45.1 C57BL/6 mice. For some experiments, mice were immunized with OVA protein (50 μg) emulsified in complete Freund’s adjuvant (Sigma-Aldrich).

### ELISA

Lymph node and spleen cells were cultured in 96-well flat-bottom plates at a concentration of 1 x 10^6^ cells/well in 0.2 ml of medium. ELISAs for INF-γ and IL–17 used 24-h supernatants using Ab pairs from e-Biosciences (La Jolla, CA). ELISA kits from e-Biosciences were used to measure IFN-γ, IL–4, and IL–17 levels in 72-h supernatants. Mice were immunized with OVA protein (50 μg) emulsified in complete Freund’s adjuvant (Sigma-Aldrich). OVA protein in PBS (50 μg/ml) was used to coat a 96 well plate overnight at 4°C. After washing with PBS + 0.1% Tween 20, serially diluted serum samples were added and incubated at room temperature for 1 hour. Wells were washed with PBS + 0.05% Tween 20, followed by adding alkaline phosphatase-conjugated goat anti-mouse IgG, IgG1, IgM, IgG2a, or IgG2c (Southern Biotech). Alkaline phosphatase activity was determined using 4-nitrophenyl phosphate disodium salt hexahydrate (Sigma-Aldrich) as the substrate.

### Western blotting

Total T cells were purified by negative selection using BioMag Goat anti-mouse IgG and anti-rat IgG (QIAGEN GmbH, Hilden, German) after 2.4G2 treatment, and incubated with a biotin-conjugated anti-CD3 mAb (2C11, 10 μg/ml, BioLegend, San Diego, USA) for 30 min on ice. Cells were then incubated with streptavidin (20 μg/ml; Sigma-Aldrich Co., St. Louis, USA) for the indicated periods of time at 37°C. For Western blotting, cells were lysed in 5 x lysis buffer containing 125mM Tris-HCl, pH 6.8, 4% SDS, 20% glycerol, 10% 2-mercaptoethanol, and 0.04% bromophenol blue. Cell lysates were resolved by SDS-PAGE, transferred to nitrocellulose membranes (ATTO Co., Tokyo, Japan), and probed with anti-ERK1/2 (Cell Signaling Technology, Danvers, USA) or anti-phospho-ERK1/2 (Cell Signaling Technology, Danvers, USA) antibodies followed by an HRP-conjugated anti-rabbit antibody (Bio-Rad Laboratories, Hercules, USA). Signals were detected using an ECL Western blotting kit (GE Healthcare, Buckinghamshire, UK) and Image Quant LAS 4000 (GE Healthcare).

### Statistical analysis

Results are given as means ± standard errors (S.E.’s). Group comparisons were by Student’s *t* test. A p-value of < 0.05 was considered significant.

## Results

### Slc3a2 deficiency does not affect T cell development in the thymus

**Fig 1:**
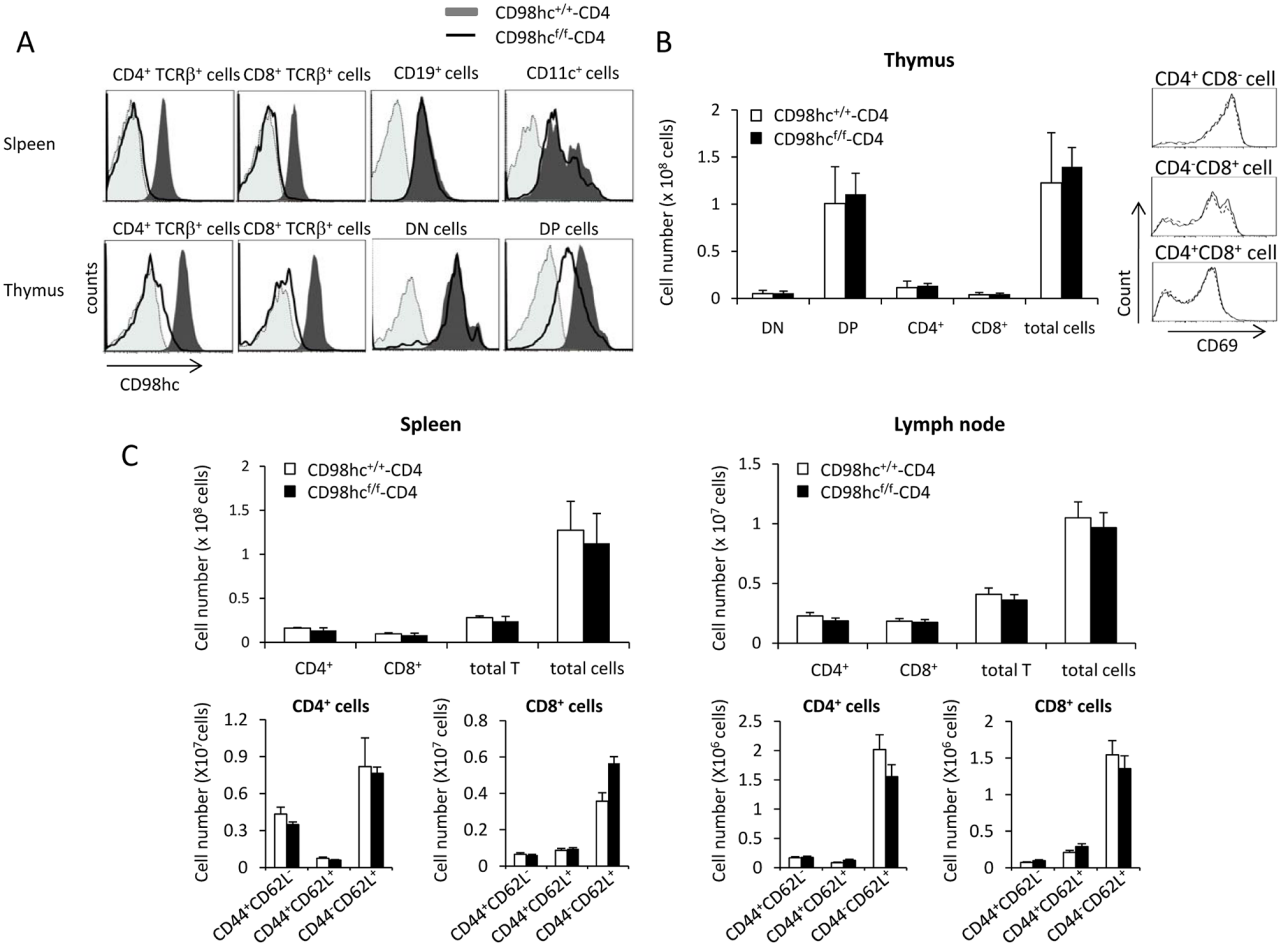
T cell development is normal in CD98hc^f/f^-CD4 mice. (A) CD4^+^TCRβ^+^, CD8^+^TCRβ^+^, CD19^+^, and CD11c^+^ cells in the spleens and CD4^+^TCRβ^+^, CD8^+^TCRβ^+^, CD4^-^CD8^-^ (DN) and CD4^+^CD8^+^ (DP) cells in the thymus of CD98hc^f/f^-CD4 mice (solid line) and CD98hc^+/+^-CD4 mice (black shadow) were stained with an anti-CD98hc mAb. CD98hc expression was evaluated by flow cytometry. Unstained cells were used as a negative control (gray shadow). (B) Thymocytes from CD98hc^f/f^-CD4 (closed) and CD98hc^+/+^-CD4 (open) mice were stained with anti-CD4 and anti-CD8 mAbs. Total cell numbers for CD4^-^CD8^-^ (DN; double negative), CD4^+^CD8^+^ (DP; double positive), CD4^+^CD8^-^ (CD4^+^), or CD4^-^CD8^+^ (CD8^+^) cells were evaluated (left). CD69 expression on CD4^+^CD8^+^, CD4^+^CD8^-^, or CD4^-^CD8^+^ cells was evaluated by flow cytometry (right). (C) Spleen cells (left) and lymph node cells (right) from CD98hc^f/f^-CD4 (closed) and CD98hc^+/+^-CD4 (open) mice were stained with anti-CD4, anti-CD8, anti-CD44, or anti-CD62L mAbs; total cells from 6 mice were counted. Results are means ± S.D. Data shown in this Figure are representative of three independent experiments.

*Slc3a2* ^flox/flox^ mice were crossed with *CD4-Cre* transgenic mice (CD98hc^f/f^-CD4 mice) to delete *Slc3a2* in T cells only. *Slc3a2* was efficiently deleted in CD4^+^TCRβ^+^ and CD8^+^TCRβ^+^ T cells, but not in CD19^+^ and CD11c^+^ cells, in the spleens of CD98hc^f/f^-CD4 mice (Fig 1A and S1 Fig). The deletion of *Slc3a2* was nearly complete in the CD4^+^TCRβ^+^ and CD8^+^TCRβ^+^ T cells in thymus (Fig 1A and S2 Fig). In contrast, the expression intensity of CD98hc on CD4^+^CD8^+^ cells in the thymus of CD98hc^f/f^-CD4 mice is about half that of control mice, suggesting that one allele of *Slc3a2* is deleted in most of the CD4^+^CD8^+^ cells and about half the amount of CD98hc protein is still present on the surface even after deletion of both alleles (Fig 1A and S2 Fig). The expression of *Slc3a2* is unaffected in TCRγδ^+^ cells from CD98hc^f/f^-CD4 mice, and about half of the CD4^-^CD8^-^TCRβ^+^ cells express CD98hc in CD98hc^f/f^-CD4 mice (S3 Fig).

We examined T cell development in the thymuses of CD98hc^f/f^-CD4 mice (Fig 1B). The total numbers of CD4^-^CD8^-^, CD4^+^CD8^+^, CD4^+^, and CD8^+^ T cells were comparable between CD98hc^f/f^-CD4 and control mice (Fig 1B). Expression of an early activation marker, CD69, on CD4^+^CD8^+^ cells, CD4^+^, and CD8^+^ cells was also comparable between CD98hc^f/f^-CD4 and control mice (Fig 1B). The total cell numbers in the spleens and lymph nodes of CD98hc^f/f^-CD4 mice were similar to those of CD98hc^+/+^-CD4 mice (Fig 1C). The expression patterns of CD44 and CD62L on CD4^+^ and CD8^+^ T cells in the spleens and lymph nodes were comparable between CD98hc^+/+^-CD4 mice and CD98hc^f/f^-CD4 mice (Fig 1C). The expression of TCRαβ and TCRγδ in CD4^+^, CD8^+^, CD4^+^CD8^+^ or CD4^-^CD8^-^ cells in the thymus was comparable between CD98hc^f/f^-CD4 and control mice (S4 Fig). Taken together, these data suggest that CD98hc does not affect T cell development in the thymus and it is not required for maintaining naïve and memory T cells in peripheral lymphoid organs.

### CD98hc is essential for T cell proliferation in vitro

**Fig 2:**
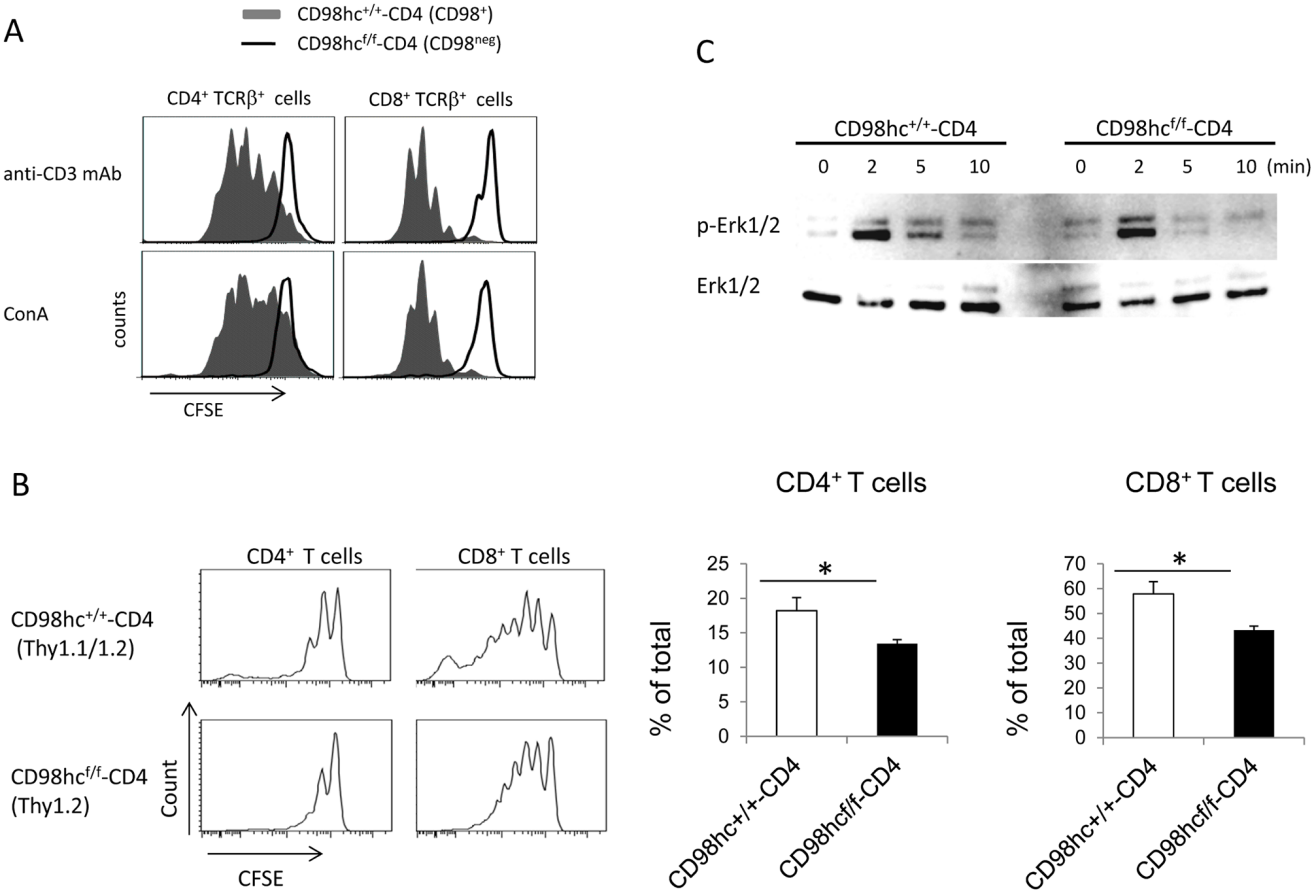
T cell proliferation is impaired in CD98hc^f/f^-CD4 mice. (A) Spleen cells from CD98hc^+/+^-CD4 (solid) or CD98hc^f/f^-CD4 (dotted) mice were labeled with CFSE and stimulated with either an anti-CD3 mAb or ConA for three days. CFSE expression after gating for CD4^+^CD98hc^+^ or CD8^+^CD98hc^+^ T cells (CD98hc^+/+^-CD4) or CD4^+^CD98hc^-^ or CD8^+^CD98hc^-^ T cells (CD98hc^f/f^-CD4) was evaluated by flow cytometry. (B) Spleen cells from CD98hc^f/f^-CD4 (Thy1.2) or CD98hc^+/+^-CD4 (Thy1.1/Thy1.2) mice were labeled with CFSE and transferred into 6 Gy-irradiated Thy1.1 C57BL/6 mice. Six days after transfer, CFSE expression after gating on CD4^+^ or CD8^+^ T cells was evaluated by flow cytometry. In the right figure, the percentage of cells that divided more than two times was calculated. Results are mean ± S.D. from 6 mice; * significant difference (*p*<0.05). (C) Purified T cells from CD98hc^f/f^-CD4 or CD98hc^+/+^-CD4 mice were stimulated with an anti-CD3 mAb for the indicated periods of time and ERK1/2 phosphorylation was determined by Western blotting. As a control, total ERK1/2 in each sample was determined. Data shown in this figure are representative of three experiments.

To determine if CD98hc affected T cell proliferation *in vitro*, splenic CD4^+^ or CD8^+^ T cells from CD98hc^f/f^-CD4 mice were labeled with CFSE and stimulated with either an anti-CD3 mAb or Con A for three days. The proliferation of CD4^+^ or CD8^+^ T cells was evaluated by CFSE dilution (Fig 2A and S5 Fig). CD98hc-deficient CD4^+^ and CD8^+^ T cells did not proliferate after stimulation with an anti-CD3 mAb or Con A (Fig 2A). To determine if CD98hc was required for T cell proliferation *in vivo*, CFSE-labeled splenic T cells from CD98hc^f/f^-CD4 (Thy1.2) or CD98hc^+/+^-CD4 (Thy1.1/Thy1.2) mice were transferred into 6 Gy-irradiated Thy1.1 B6 mice. In contrast to the complete abrogation of T cell proliferation *in vitro*, both CD4^+^ and CD8^+^ T cells could proliferate although the proliferative responses were weaker for CD4^+^ and CD8^+^ T cells from CD98hc^f/f^-CD4 mice than from CD98hc^+/+^-CD4 mice (Fig 2B). These data indicate that CD98hc plays a crucial role in T cell proliferation both *in vitro* and *in vivo*.

To determine the mechanism for impaired T cell proliferation in CD98hc^f/f^-CD4 mice, we assessed ERK1/2 phosphorylation after anti-CD3 mAb-mediated stimulation. The peak for ERK1/2 phosphorylation at 2 min after stimulation was comparable between CD98hc negative and control T cells, while ERK1/2 phosphorylation declined faster in CD98hc negative cells than in control T cells (Fig 2C). These data suggested that CD98hc was required for maintaining ERK1/2 phosphorylation.

### Antigen-specific antibody production is absent in CD98hc^f/f^-CD4 mice.

**Fig 3:**
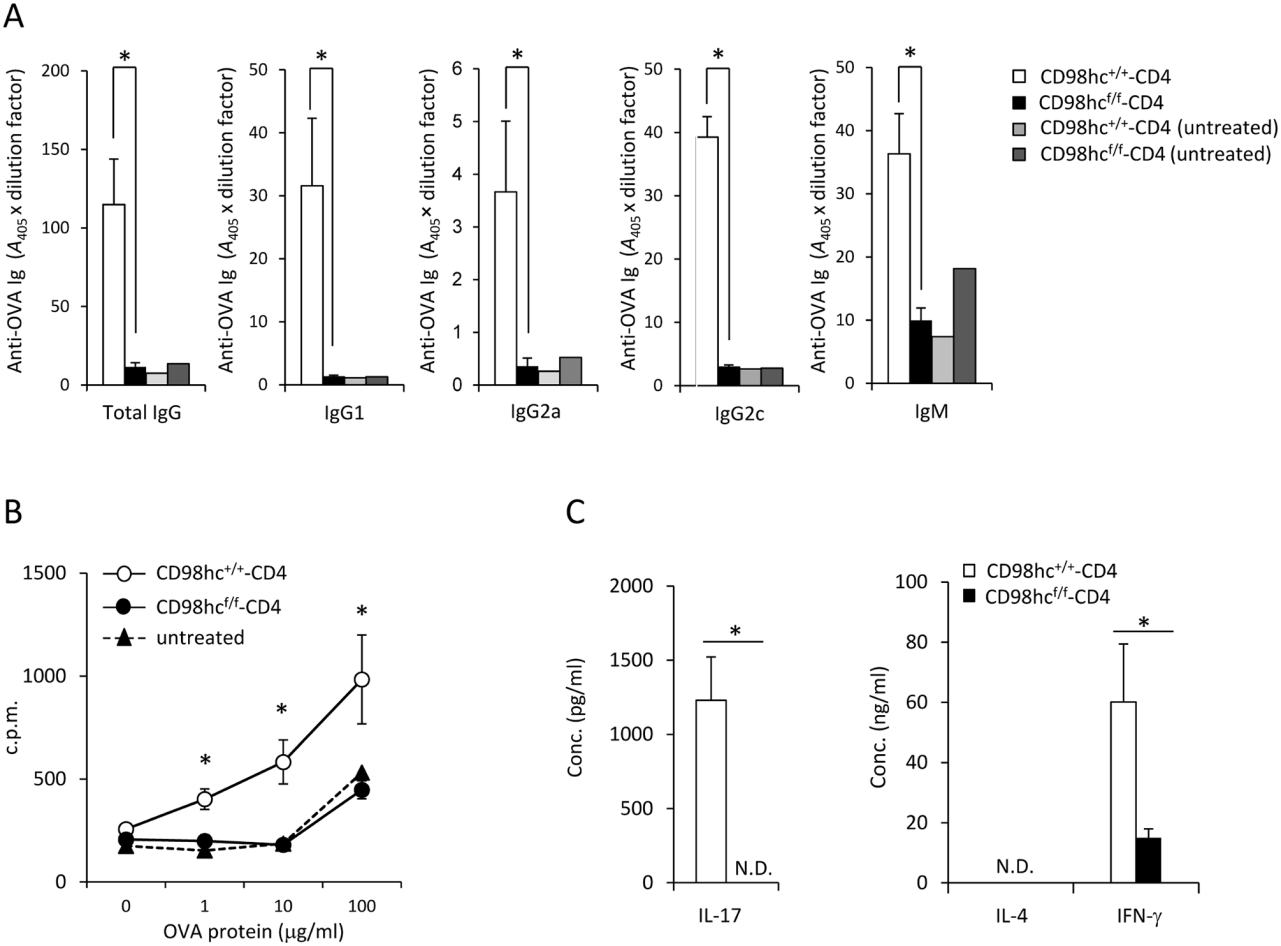
Functional differentiation of CD4^+^ T cells is impaired in CD98hc^f/f^-CD4 mice. CD98hc^f/f^-CD4 or CD98hc^+/+^-CD4 mice were immunized with OVA protein emulsified in CFA. (A) Serum anti-OVA specific IgG, IgG1, IgG2a, IgG2c, and IgM titers at eight days after OVA immunization were determined by ELISA. As negative controls, sera from unimmunized CD98hc^f/f^-CD4 and CD98hc^+/+^-CD4 mice were used. Results are means ± S.D. of 7 mice; * significant difference (*p*<0.05). (B) Total lymph node cells from OVA-immunized CD98hc^f/f^-CD4 or CD98hc^+/+^-CD4 mice were stimulated with OVA protein for 3 days. [^3^H]-thymidine incorporation during the final 6 hours was determined. Results are means ± S.D. of 5 mice; * significant difference (*p*<0.05). (C) After stimulating T cells from OVA immunized CD98hc^f/f^-CD4 and CD98hc^+/+^-CD4 mice with OVA protein (50 μg/ml) for 3 days, culture supernatant concentrations of IL–4, IL–17, and IFN-γ were determined by ELISA. Results are means ± S.D. of 3 mice; * significant difference (*p*<0.05). Data shown in this Figure are representative of three experiments.

To test if T cell-specific immune responses were affected by CD98hc deficiency, CD98hc^f/f^-CD4 mice were immunized with OVA protein emulsified in CFA, after which OVA-specific antibody production was determined. CD98hc^f/f^-CD4 mice could not produce anti-OVA specific IgM, IgG, IgG1, IgG2a, or IgG2c (Fig 3A). Anti-OVA IgG was not detected even one month after immunization in CD98hc^f/f^-CD4 mice (data not shown). T cells from OVA-immunized CD98hc^f/f^-CD4 mice could not proliferate when stimulated with OVA protein as compared with the antigen-dependent proliferation of T cells from CD98hc^+/+^-CD4 mice (Fig 3B). The expression patterns of activation markers such as CD44, CD62L, CD25 and CD69 in CD4^+^ and CD8^+^ T cells 8 days after immunization were comparable between CD98hc^f/f^-CD4 and control mice (S6 Fig). IL–17 and IL–4 secretion was not detected by T cells from OVA-immunized CD98hc^f/f^-CD4 mice after stimulation with OVA protein *in vitro* (Fig 3C). IFN-γ secretion was significantly reduced for T cells from CD98hc^f/f^-CD4 mice compared to T cells from CD98hc^+/+^-CD4 mice (Fig 3C). These data indicate that CD98hc deficiency in T cells impairs T cell proliferation and Th1 and Th17 differentiation at the cell population level.

### Th1 differentiation is impaired in CD98hc^f/f^-CD4 mice.

**Fig 4:**
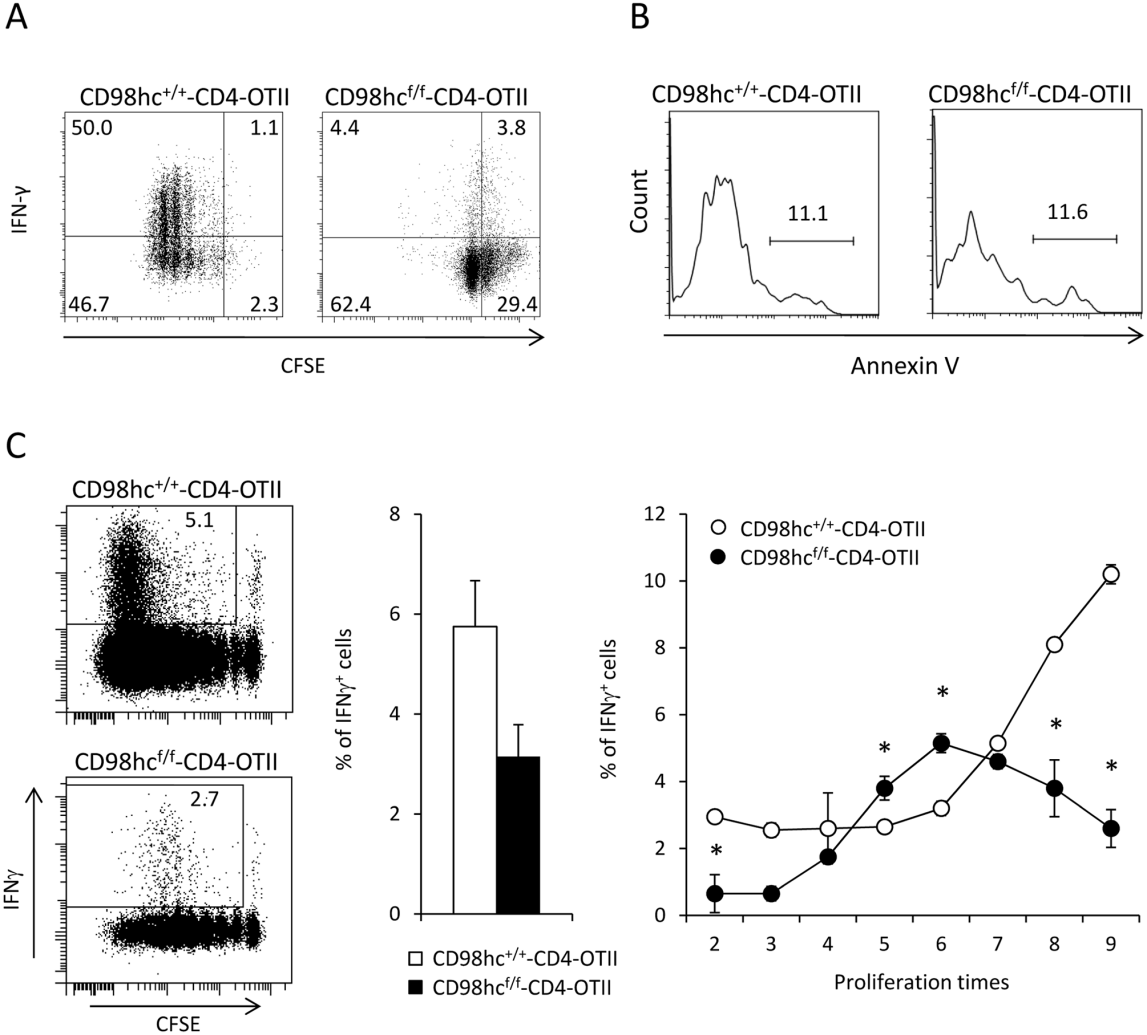
IFN-γ secretion is disturbed in CD98hc^f/f^-CD4 mice. (A) CFSE labeled spleen cells from CD98hc^f/f^-CD4-OT11 or CD98hc^+/+^-CD4-OT11 mice were stimulated with OVA peptides under Th1 culture conditions for 3 days. Cells were stained with anti-CD4, anti-CD98hc and anti-IFN-γ mAbs and evaluated by flow cytometry. Data shown are gated on CD98hc^-^ cells. (B) Spleen cells from CD98hc^f/f^-CD4-OT11 or CD98hc^+/+^-CD4-OT11 mice were stimulated with OVA peptides under Th1 culture conditions for 3 days. Cells were stained with anti-CD4 and AnnexinV, and evaluated by flow cytometry. (C) Cells from CD98hc^f/f^-CD4-OT11 (Thy1.2) or CD98hc^+/+^-CD4-OT11 (Thy1.1^+^/Thy1.2^+^) mice were labeled with CFSE and transferred into CD45.1 C57BL/6 mice. Mice were then immunized with OVA protein. CFSE dilution and intracellular INF-γ expression were evaluated by flow cytometry gated on CD98hc^+^ (CD98hc^+/+^-CD4-OT11) or CD98^-^ cells (CD98hc^f/f^-CD4-OT11) cells four days after immunization (left). The percentages of INF-γ^+^ cells among total CD4^+^ T cells are shown (middle). The percentages of INF-γ^+^ cells among total CD4^+^ cells that underwent cell division at the indicated times were counted (right). Results are means ± S.D. of 4 mice; * significant difference (*p*<0.05). Data shown in this Figure are representative of three experiments.

One study of different CD98hc^f/f^ mice crossed with *lck-Cre* transgenic mice showed that CD98hc was not intrinsically required for effector functions of CD4^+^ and CD8^+^ T cells [12]. However, it remains unclear whether CD98hc directly regulates effector function independent of T cell proliferation. To evaluate the roles of CD98hc in Th1 differentiation, cell division, and T cell survival, CFSE-labeled CD4^+^ T cells from CD98hc^f/f^-CD4-OT11 or CD98hc^+/+^-CD4-OT11 mice were stimulated with OVA peptides under Th1 condition. CD4^+^ T cells from CD98hc^f/f^-CD4-OT11 mice showed reduced proliferation and less IFN-γ production than CD4^+^ T cells from CD98hc^+/+^-CD4-OT11 mice (Fig 4A). The relative percentages of IFN-γ producing cells among undivided cells or divided cells were lower in CD98hc^f/f^-CD4-OT11 than in CD98hc^+/+^-CD4-OT11 mice (Fig 4A). Because reduced cell division or IFN-γ production in CD98hc^f/f^-CD4-OT11 mice might be attributable to increased cell death, we stained CD4^+^ T cells from CD98hc^f/f^-CD4-OT11 and CD98hc^+/+^-CD4-OT11 mice with annexin V during Th1 differentiation. The percentages of annexin V-positive cells were comparable between these two groups, which suggested that CD98hc deficiency did not intrinsically affect T cell survival (Fig 4B). To determine the relationship between T cell proliferation and cytokine production *in vivo*, we labeled CD4^+^ T cells from CD98hc^f/f^-CD4-OT11 (Thy1.1^-^Thy1.2^+^ and CD45.2^-^CD45.2^+^) or CD98hc^+/+^-CD4-OT11 (Thy1.1^+^Thy1.2^+^ and CD45.1^-^CD45.2^+^) mice with CFSE and transferred these cells into CD45.1 C57BL/6 mice (CD45.2^+^CD45.2^-^) (Fig 4C and S7 Fig). Then, mice were immunized with OVA protein, after which CFSE dilution and intracellular INF-γ expression were evaluated. CD4^+^ T cells from CD98hc^f/f^-CD4-OT11 mice divided more slowly than did cells from CD98hc^+/+^-CD4-OT11 mice at four days after immunization. There were fewer IFN-γ secreting cells from CD98hc^f/f^-CD4-OT11 mice than from control mice. Importantly, the expression intensity of INF-γ was much lower among cells from CD98hc^f/f^-CD4-OT11 mice than those from CD98hc^+/+^-CD4-OT11 mice, even among cells that divided at the same time. These data indicate that CD98hc deficiency disrupts INF-γ secretion at least partly independently of cell division.

### CD98^f/f^-CD4 mice cannot control *Leishmania major* infection.

**Fig 5:**
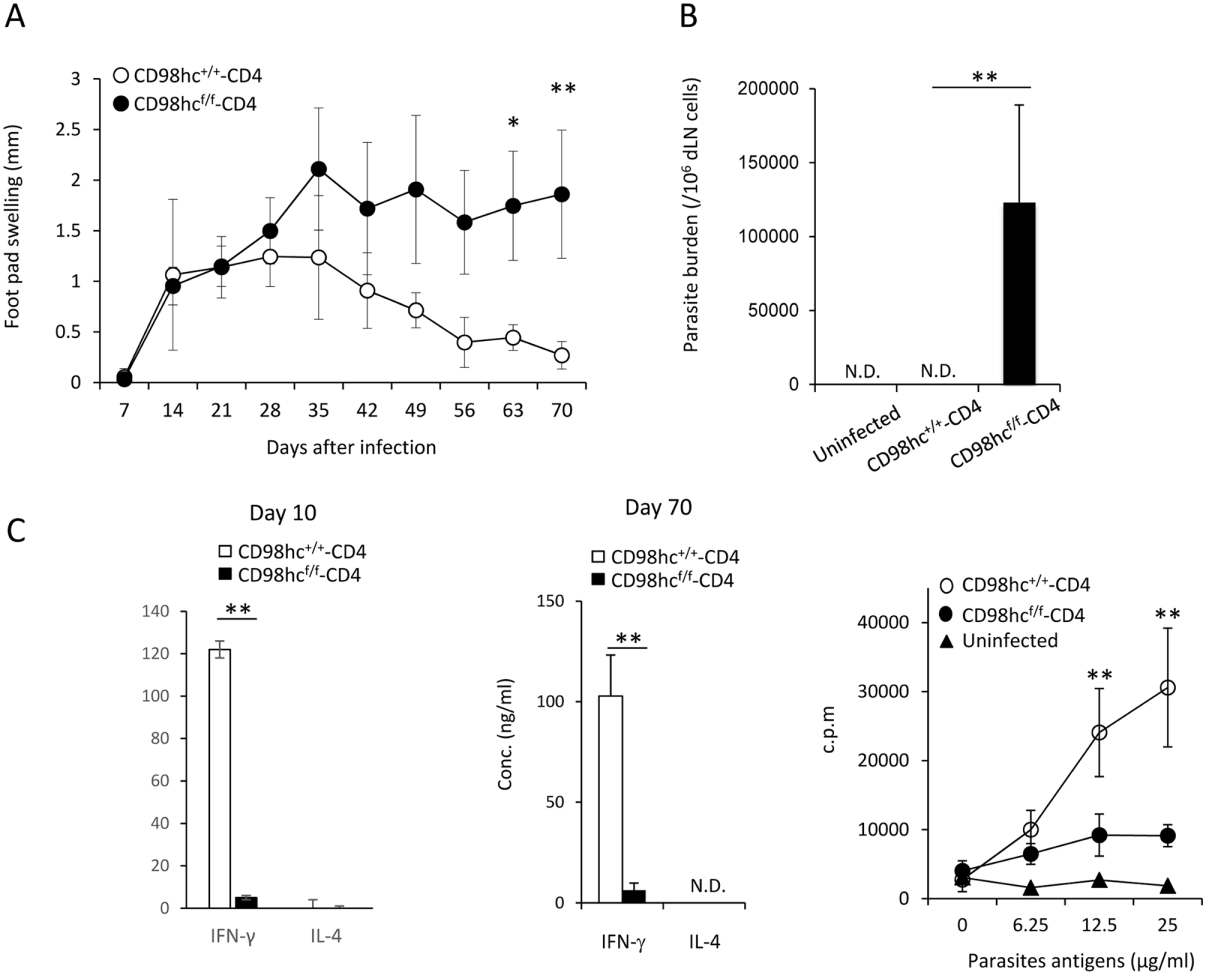
T cells from CD98hc^f/f^-CD4 mice cannot mount immune responses against-*Leishmania major*. CD98hc^+/+^-CD4 or CD98hc^f/f^-CD4 mice under a C57BL/6 background were infected with *Leishmania majo*r in the footpad. (A) Footpad swelling in CD98hc^+/+^-CD4 (closed) or CD98hc^f/f^-CD4 (open) mice was measured after infection. Results are means ± S.D. of 8 mice; *or ** significant difference (*p* < 0.05 or p < 0.01, respectively). (B) Total popliteal lymph nodes from *Leishmania major*-infected CD98hc^+/+^-CD4 or CD98hc^f/f^-CD4 mice (day 30) were cultured for 5 days. Then, parasite numbers were counted and parasites/lymph node cells were determined. Results are means ± S.D. of 8 mice; ** significant difference (*p*<0.01). (C) Purified CD4^+^ T cells (5 x 10^5^/well) from lymph node cells from CD98hc^f/f^-CD4 (closed) or CD98hc^+/+^-CD4 (open) mice infected with *Leishmania major* (day 10 and day 70) were stimulated with irradiated spleen cells (2 x 10^5^/well) and parasite-derived antigens for 3 days. Then, IFN-γ and IL–4 in culture supernatants were determined by ELISA. Results are means ± S.D. of 8 mice; * or ** significant difference (*p*<0.05 or p < 0.01, respectively). Purified CD4^+^ T cells (5 x 10^5^/well) from lymph node cells from CD98hc^f/f^-CD4 (closed circle) or CD98hc^+/+^-CD4 (open circle) mice infected with *Leishmania major* (day 20) were stimulated with irradiated spleen cells (2 x 10^5^/well) and parasite-derived antigens for 3 days. Then, [^3^H]-thymidine incorporation was determined. Results are means ± S.D. from 8 mice; ** significant difference (*p*<0.01). Data shown in this Figure are representative of three experiments.

C57BL/6 mice respond differently to infection with *Leishmania major* parasites compared with BALB/c mice, as C57BL/6 mice can remove these parasites through Th1 responses and activated macrophages [17] [18]. We next examined the contribution of CD98hc on effector T cell differentiation in *Leishmania major* infection in CD98hc^f/f^-CD4 mice. CD98hc^+/+^-CD4 and CD98hc^f/f^-CD4 mice (C57BL/6 background) were infected with *Leishmania major* parasites in their foot pads, after which parasite load and disease severity were monitored. The control CD98hc^+/+^-CD4 mice could readily control *Leishmania major* infection, while CD98hc^f/f^-CD4 mice were relatively susceptible to this infection because their foot pad swelling increased compared with CD98^+/+^-CD4 mice (Fig 5A).

We measured the parasite numbers in CD98hc^f/f^-CD4 mice and CD98hc^+/+^-CD4 mice infected with *Leishmania major* 70 days after infection (Fig 5B). Parasites were not detected in CD98hc^+/+^-CD4 mice, while large numbers of parasites were still present in the foot pads of CD98hc^f/f^-CD4 mice (Fig 5B). Differentiation of CD4^+^ T cells toward Th1 cells is required for controlling *Leishmania major* infection. CD4^+^ T cells from *Leishmania major* infected CD98hc^f/f^-CD4 and CD98hc^+/+^-CD4 mice 10 or 70 days after infection were stimulated with *Leishmania major*-derived antigens for three days and IFN-γ levels in culture supernatants were measured (Fig 5C). CD4^+^ T cells from CD98hc^f/f^-CD4 mice secreted little IFN-γ and exhibited minimal T cell proliferation after stimulation with parasite antigens at both 10 and 70 days after infection (Fig 5C). T cell proliferation against parasite-derived antigens 70 days after infection was observed in CD98hc^f/f^-CD4 mice while strong responses in control mice (Fig 5C). We have compared the activation markers and Foxp3^+^ regulatory T cell numbers in control and CD98hc^f/f^-CD4 mice and did not find any difference (data not shown). These data indicate that CD98hc is required for controlling *leishmania major* infection by inducing IFN-γ production.

## Discussion

A variety of molecules are involved in the functional differentiation of CD4^+^ T cells [1] [19] [3] [20]. The proper functional differentiation of CD4^+^ T cells is essential for defenses against pathogens and is accompanied by T cell proliferation [1] [3] [20]. We demonstrated that CD98hc was important for Th1 differentiation, as assessed by INF-γ secretion, together with T cell proliferation. *Slc3a2*-deficient mice could not mount efficient Th1 responses against *Leishmania major* infection. By carefully analyzing the association between IFN-γ production and CD4^+^ T cell division, IFN-γ production *in vivo* was impaired by deleting CD98hc from CD4^+^ T cells. These data indicate that CD98hc is crucial for Th1 differentiation, which is at least partly independent of T cell proliferation.

A previous study using *Slc3a2* floxed mice that were from a different strain than those used in our experiments also showed that T cell proliferation was disturbed by CD98hc deficiency in T cells [12]. Furthermore, that study indicated that T cell effector functions were not intrinsically impaired by CD98hc deficiency, although the total Th1 response was impaired along with reduced T cell proliferative activity [12]. Therefore, in this study, we analyzed if Th1 responses in CD98hc deficient mice were impaired *in vivo*. We crossed *Slc3a2* floxed mice with *CD4-Cre* transgenic mice rather than the *lck-Cre* transgenic mice that were used in the previous study [12]. Because T cell proliferation is a critical factor for the acquisition of effector functions, we evaluated the association of IFN-γ production by CD4^+^ T cells and cell division *in vivo*. We found reduced IFN-γ production by CD98hc deficient CD4^+^ T cells that had undergone the same number of cell divisions as control CD4^+^ T cells, indicating the intrinsic necessity of CD98hc for Th1 differentiation. At 4 or 5 cell divisions, the relative number of IFN-γ producers was higher in CD98hc-deficient T cells. It could be attributable to the lower proliferative ability of CD98hc-deficient IFN-γ-producing T cells. In addition, CD98hc-deficient T cells were not more prone to undergo apoptosis compared with control T cells under Th1 culture conditions. We also tested if CD98hc deficiency in T cells altered the susceptibility to *Leishmania major* parasites because Th1 responses are crucial for controlling this infection. Our data indicated that C57BL/6 mice with T cells deficient in CD98hc failed to control a *Leishmania major* infection associated with reduced Th1 responses. Taken together, these data strongly suggest that IFN-γ production is intrinsically impaired by CD98hc deficiency in CD4^+^ T cells, which is at least partly independent of T cell proliferation and apoptosis.

CD4^+^ and CD8^+^ T cell proliferation *in vitro* was completely abolished using cells from our *Slc3a2*-deficient mice, while the proliferative ability of T cells from other *Slc3a2*-deficient mice after TCR ligation was present, although it was reduced compared to control T cells [12]. In contrast, in *in vivo* experiments, our and other *Slc3a2*-deficient mice exhibited a partial defect in T cell proliferation [12]. Either exon 3 or exons 1 and 2 of *Slc3a2* were deleted in our strain or another strain, respectively, which might have contributed to the observed differences, although CD98hc expression was almost completely abolished in both strains. Another difference was the use of *Cre* transgenic mice to delete *Slc3a2*. We used *CD4-Cre*, while another group used *lck-Cre* transgenic mice [12]. The timing for deleting *Slc3a2* during an immature T cell stage might affect the proliferative capacity of descendant T cells. In any event, in both strains, T cell proliferative responses were weaker than control T cells *in vivo*, which lead to the same conclusion that CD98hc is crucial for T-cell mediated adaptive immune responses. We also found that TCR-mediated ERK1/2 phosphorylation declined more rapidly in CD98hc negative T cells than in control T cells. Because ERK1/2 phosphorylation is important for T cell proliferation [21] [22], the rapid decline of ERK1/2 phosphorylation might contribute to the lower proliferative activity of CD98hc-deficient T cells, although it is necessary to examine the molecular interaction of CD98hc and ERK phosphorylation in future studies.

Our group has previously demonstrated that an anti-CD98hc blocking antibody had a therapeutic effect on spontaneously developed type 1 diabetes in NOD mice [13]. Another group also showed that T cell-specific CD98hc deficiency prevented the development of type1 diabetes and experimental autoimmune encephalomyelitis [12]. These data suggest that blocking CD98hc might be a useful strategy for treating T cell-mediated autoimmune disorders. However, CD98hc is also expressed by non-immune cells. Therefore, it would be important to block CD98hc functions in immune cells only to establish a therapeutic strategy based on blocking CD98hc functions for treating immune-mediated disorders.

## Supporting Information

S1 Fig Gating strategy of spleen cells used for flow cytometry analysis. Spleen cells were first analyzed based on their forward scatter and side scatter profiles. Viable cells were gated based on negative staining for 7-AAD. 7-AAD negative cells were gated on the CD4^+^TCRβ^+^, CD8^+^TCRβ^+^, CD19^+^TCRβ^-^ or CD11c^+^TCRβ^-^ population.
(PDF)

S2 Fig Gating strategy of thymocytes used for flow cytometry analysis. Thymocytes were first analyzed based on their forward scatter and side scatter profiles. Viable cells were gated based on negative staining for 7-AAD. Cells were stained with anti-CD4, CD8 and TCRβ antibodies.
(PDF)

S3 Fig CD98hc expression in CD98hc^f/f^-CD4 mice. Cells were first analyzed based on their forward scatter and side scatter profiles. Viable cells were gated based on negative staining for 7-AAD. CD4^-^CD8^-^TCRβ^+^ and TCRγδ^+^ spleen cells from CD98hc^f/f^-CD4 mice or CD98hc^+/+^-CD4 mice were stained with anti-CD98hc mAb. CD4^-^CD8^-^TCRβ^+^ were further stained with anti-NK1.1 antibody. CD98hc expression was evaluated by flow cytometry. Cells stained with an isotype control antibody were used as a negative control.
(PDF)

S4 Fig Expression of TCRαβ and TCRγδ in thymic T cells. Cells were first analyzed based on their forward scatter and side scatter profiles. Viable cells were gated based on negative staining for 7-AAD. CD4^+^, CD8^+^, CD4^+^CD8^+^ or CD4^-^CD8^-^ thymus cells from CD98hc^f/f^-CD4 mice or CD98hc^+/+^-CD4 mice were stained with anti- TCRαβ and anti-TCRγδ mAbs and their expression was evaluated by flow cytometry. Cells stained with an isotype control antibody were used as a negative control.
(PDF)

S5 Fig Gating strategy of CD98hc expression used for flow cytometry analysis. Cells were first analyzed based on their forward scatter and side scatter profiles. Viable cells were gated based on negative staining for 7-AAD. Cells were stained with anti-CD4, CD8 and CD98hc antibodies.
(PDF)

S6 Fig Expression of T cell activation markers in mice immunized with OVA. CD98hc^f/f^-CD4 or CD98hc^+/+^-CD4 mice were immunized with OVA protein emulsified in CFA. Draining lymph node cells were stained with anti-CD4, anti-CD8, anti-CD25, anti-CD69, anti-CD44, and anti-CD62L antibodies. The expression of these activation markers on CD4 and CD8 T cells was evaluated by flow cytometry.
(PDF)

S7 Fig Gating strategy of IFN-γ expression used for flow cytometry analysis. Cells were first analyzed based on their forward scatter and side scatter profiles. Cells were stained with anti-CD45.2, CD45.1, Thy1.2, Thy1.1 and CD4 antibodies and then stained by anti-IFN-γ antibody.
(PDF)
