# Supplementary figures and images for: CD98 Heavy Chain Is a Potent Positive Regulator of CD4+ T Cell Proliferation and Interferon-γ Production In Vivo
Source: PLoS One. 2015 Oct 7;10(10):e0139692. doi: 10.1371/journal.pone.0139692 (PMC4596652; doi:10.1371/journal.pone.0139692)

**S1 Fig.**

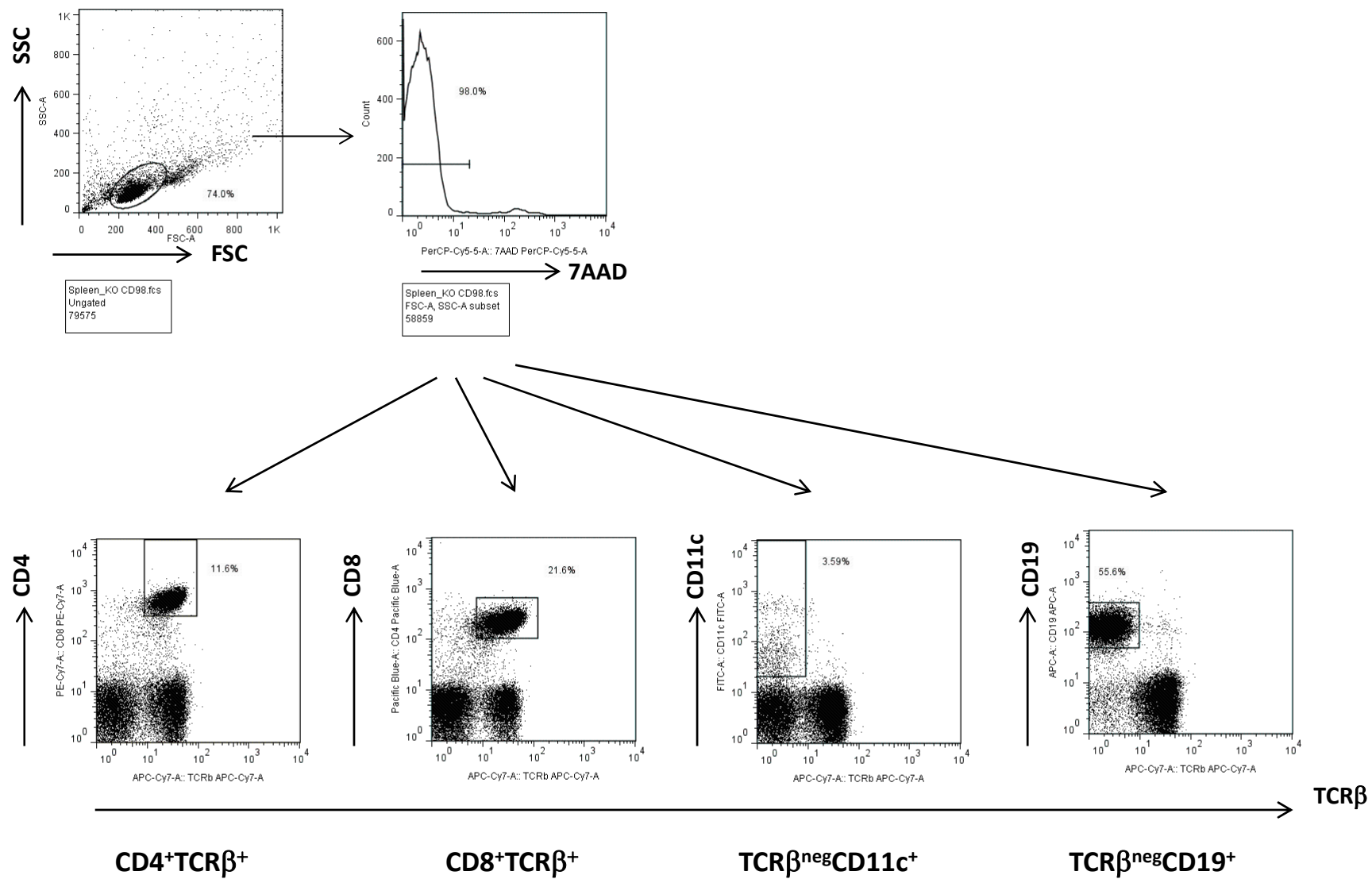

Supplement: S1 Fig — Spleen cells were first analyzed based on their forward scatter and side scatter profiles. Viable cells were gated based on negative staining for 7-AAD. 7-AAD negative cells were gated on the CD4+TCRβ+, CD8+TCRβ+, CD19+TCRβ- or CD11c+TCRβ- population. (PDF) [file pone.0139692.s001.pdf]

**S2 Fig.**

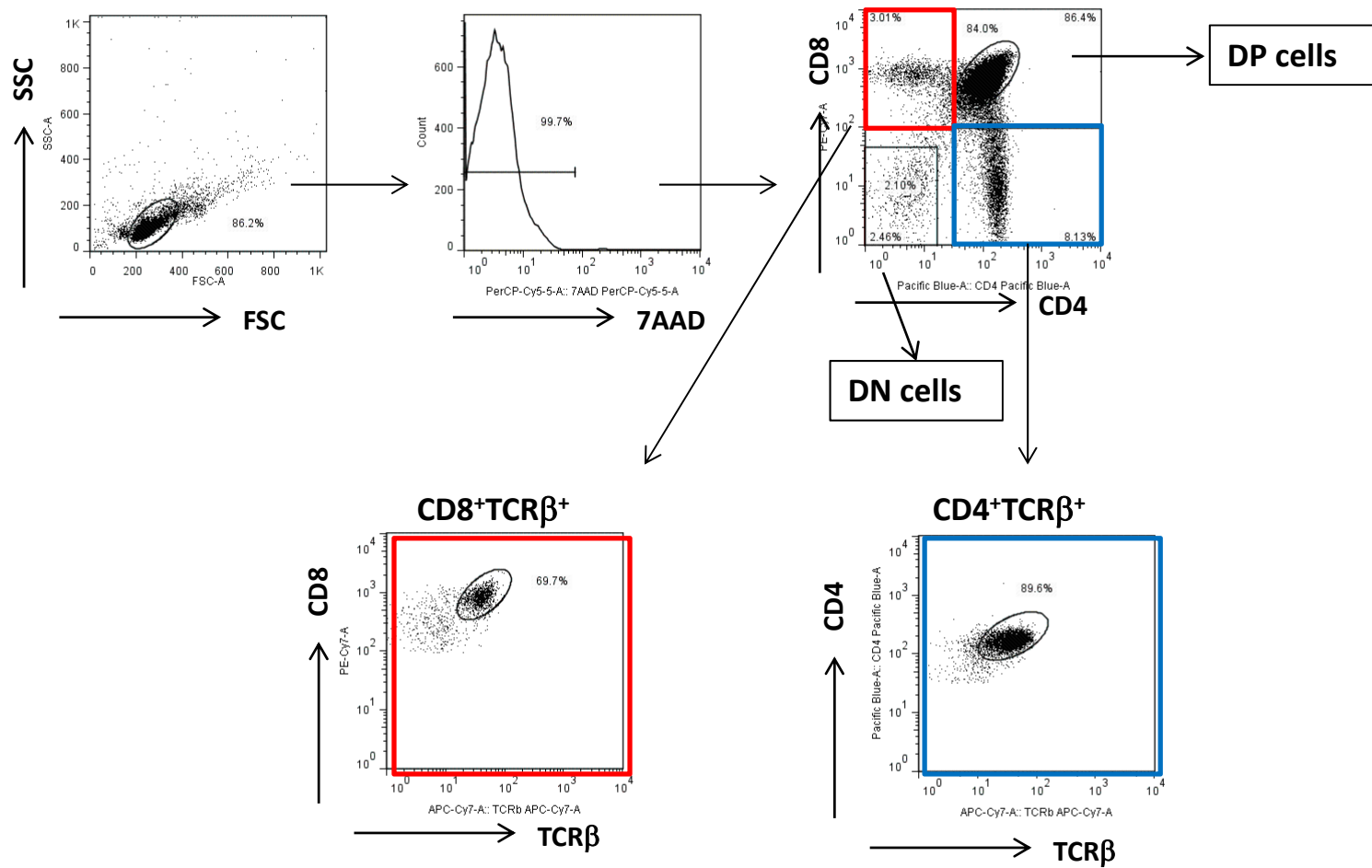

Supplement: S2 Fig — Thymocytes were first analyzed based on their forward scatter and side scatter profiles. Viable cells were gated based on negative staining for 7-AAD. Cells were stained with anti-CD4, CD8 and TCRβ antibodies. (PDF) [file pone.0139692.s002.pdf]

**S3 Fig.**

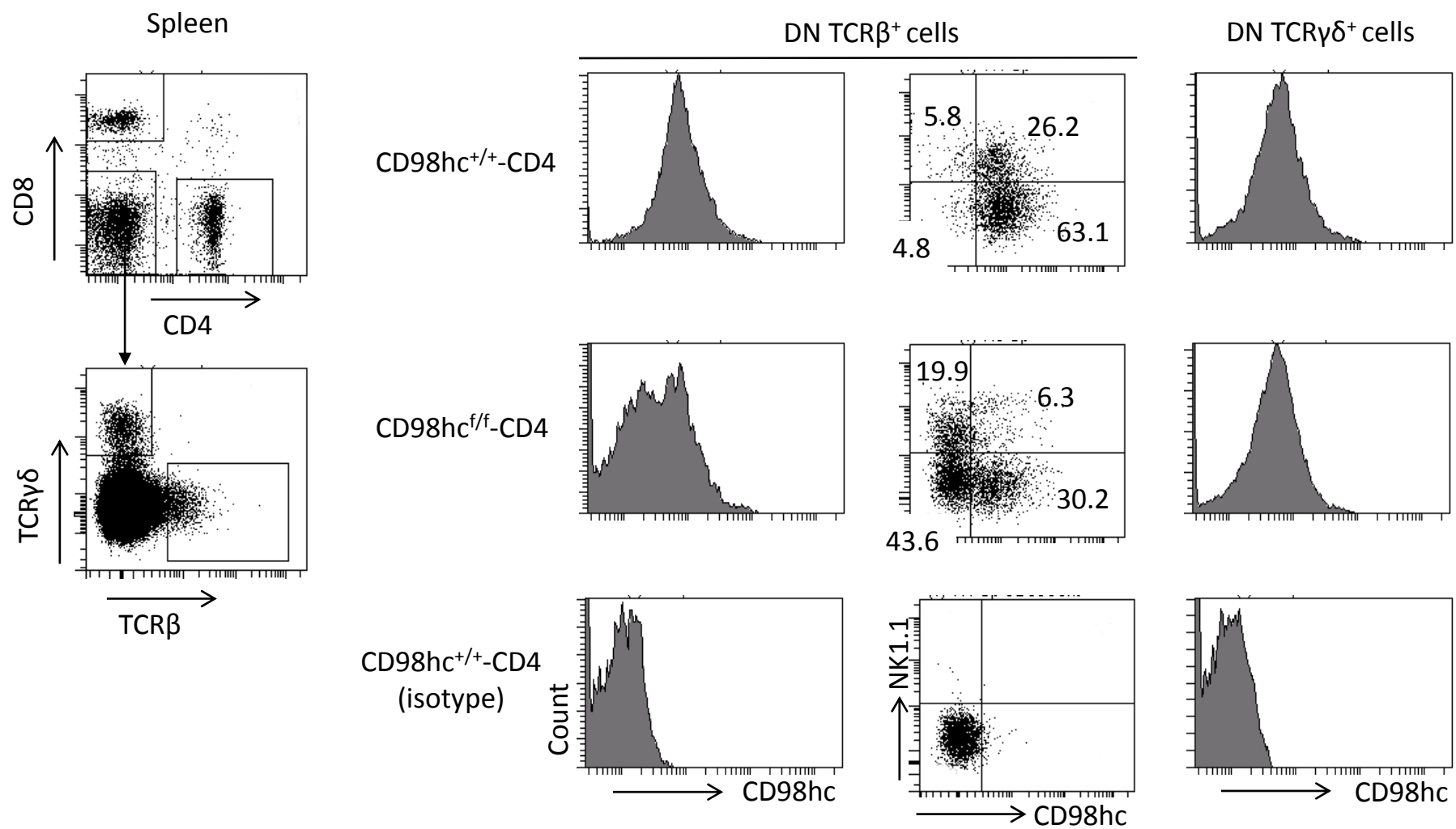

Supplement: S3 Fig — Cells were first analyzed based on their forward scatter and side scatter profiles. Viable cells were gated based on negative staining for 7-AAD. CD4-CD8-TCRβ+ and TCRγδ+ spleen cells from CD98hcf/f-CD4 mice or CD98hc+/+-CD4 mice were stained with anti-CD98hc mAb. CD4-CD8-TCRβ+ were further stained with anti-NK1.1 antibody. CD98hc expression was evaluated by flow cytometry. Cells stained with an isotype control antibody were used as a negative control. (PDF) [file pone.0139692.s003.pdf]

**S4 Fig.**

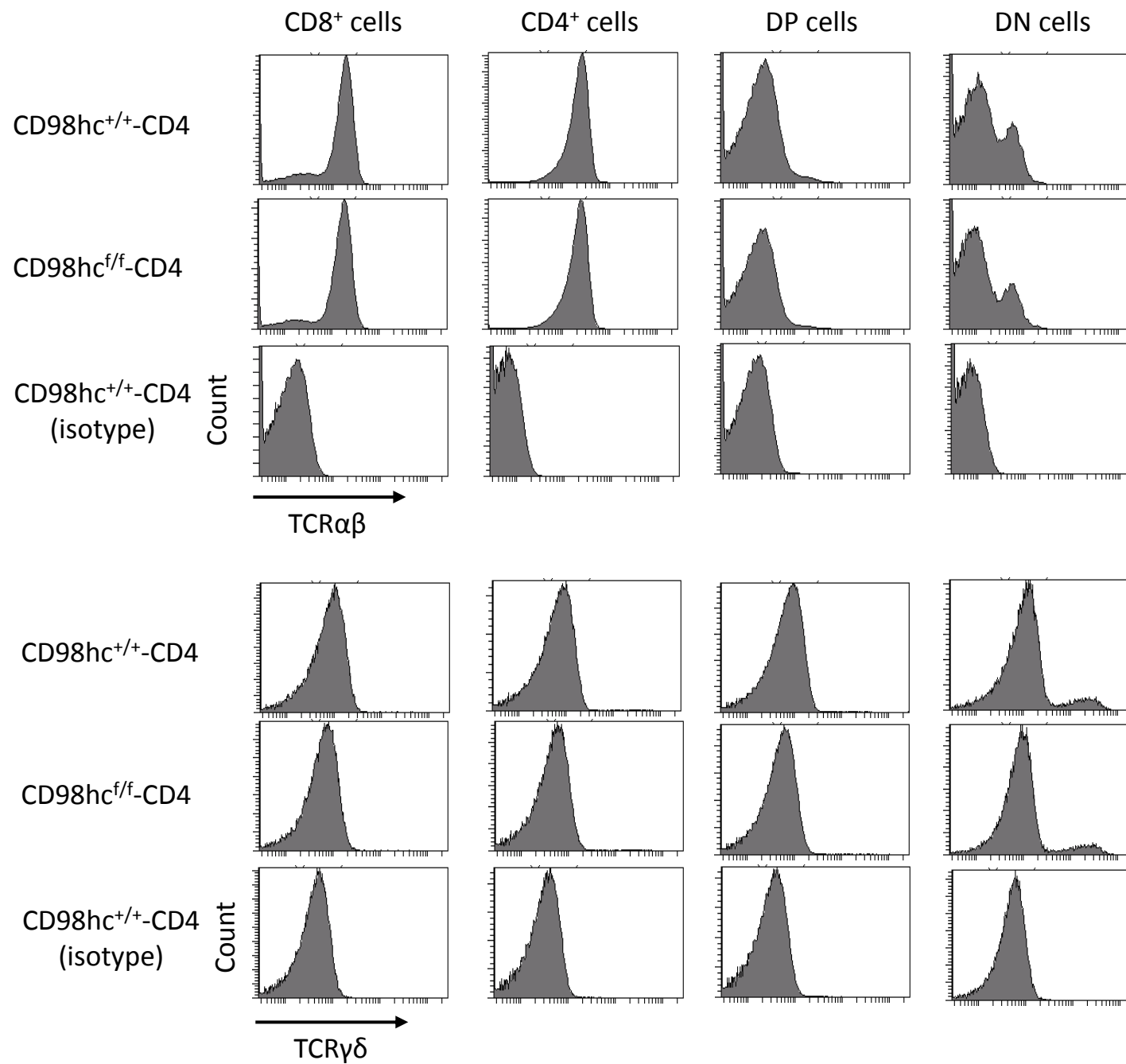

Supplement: S4 Fig — Cells were first analyzed based on their forward scatter and side scatter profiles. Viable cells were gated based on negative staining for 7-AAD. CD4+, CD8+, CD4+CD8+ or CD4-CD8- thymus cells from CD98hcf/f-CD4 mice or CD98hc+/+-CD4 mice were stained with anti- TCRαβ and anti-TCRγδ mAbs and their expression was evaluated by flow cytometry. Cells stained with an isotype control antibody were used as a negative control. (PDF) [file pone.0139692.s004.pdf]

S5 Fig.

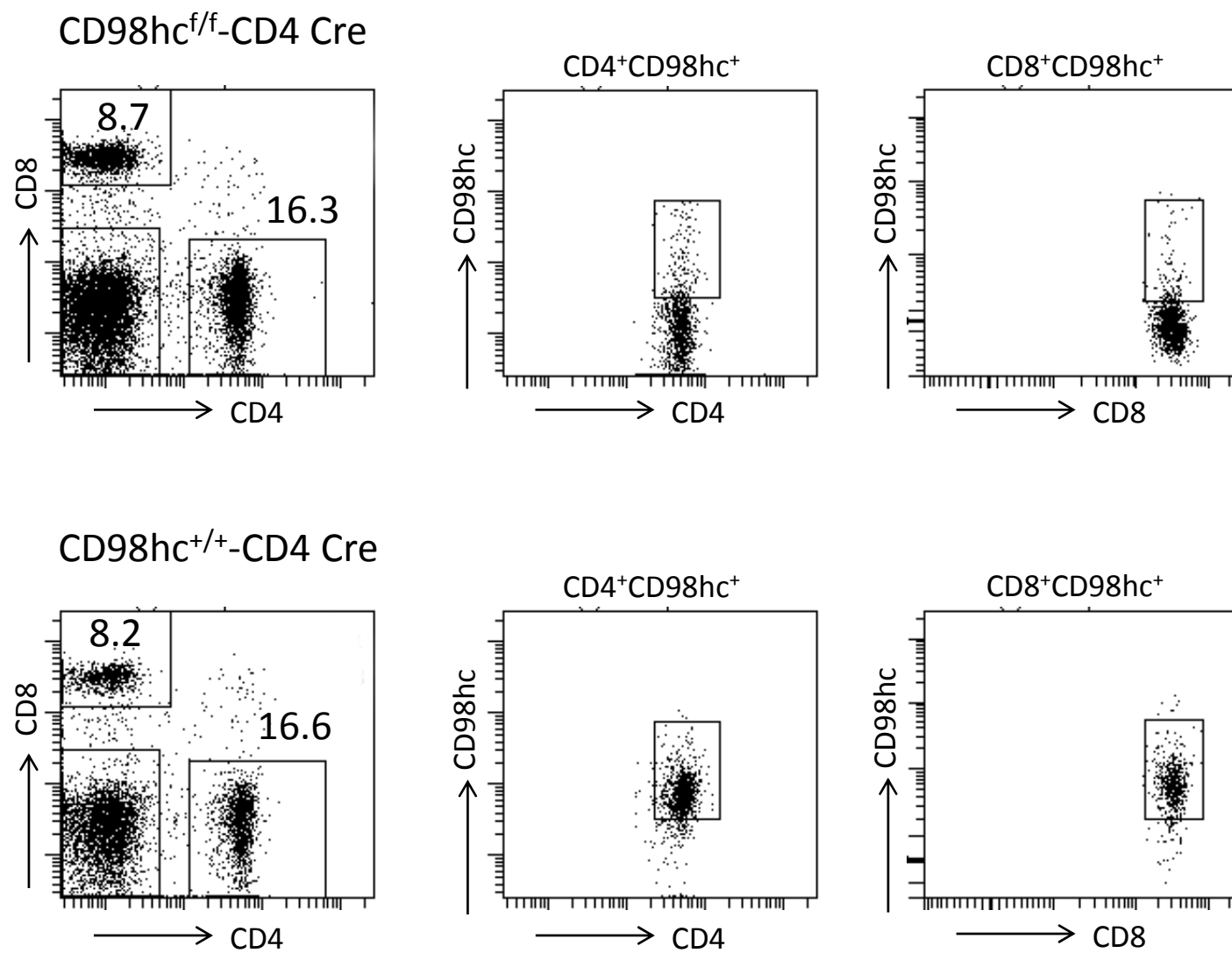

Supplement: S5 Fig — Cells were first analyzed based on their forward scatter and side scatter profiles. Viable cells were gated based on negative staining for 7-AAD. Cells were stained with anti-CD4, CD8 and CD98hc antibodies. (PDF) [file pone.0139692.s005.pdf]

S6 Fig.

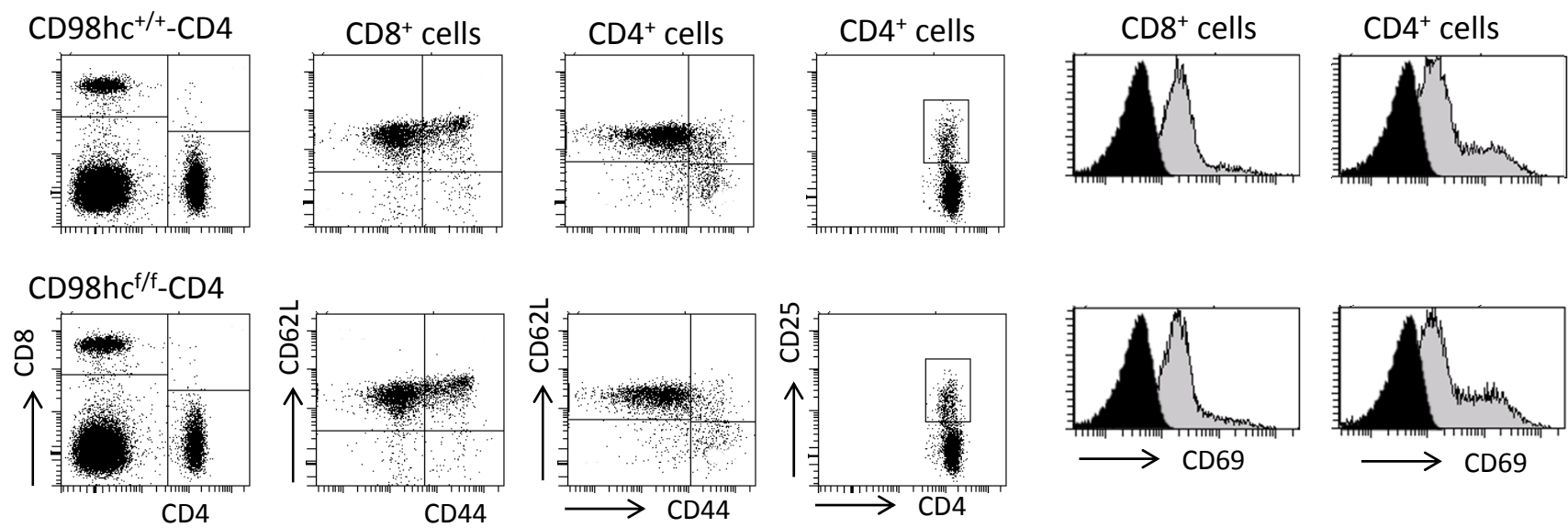

Supplement: S6 Fig — CD98hcf/f-CD4 or CD98hc+/+-CD4 mice were immunized with OVA protein emulsified in CFA. Draining lymph node cells were stained with anti-CD4, anti-CD8, anti-CD25, anti-CD69, anti-CD44, and anti-CD62L antibodies. The expression of these activation markers on CD4 and CD8 T cells was evaluated by flow cytometry. (PDF) [file pone.0139692.s006.pdf]

**S7 Fig.**

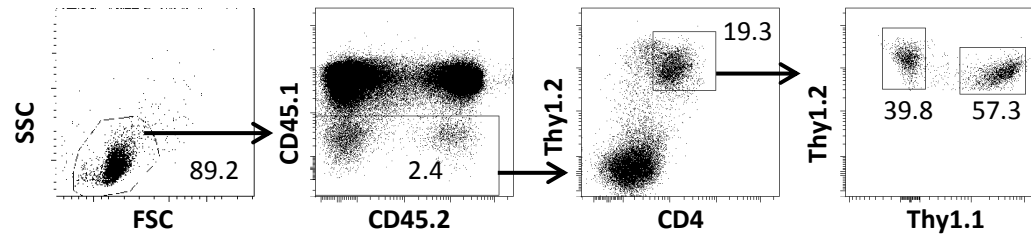

**Donor:**  
 CD98hc<sup>+/+</sup>-CD4-OTII (Thy1.1/1.2, CD45.2) → **Recipient:**  
 CD98hc<sup>f/f</sup>-CD4-OTII (Thy1.2, CD45.2)

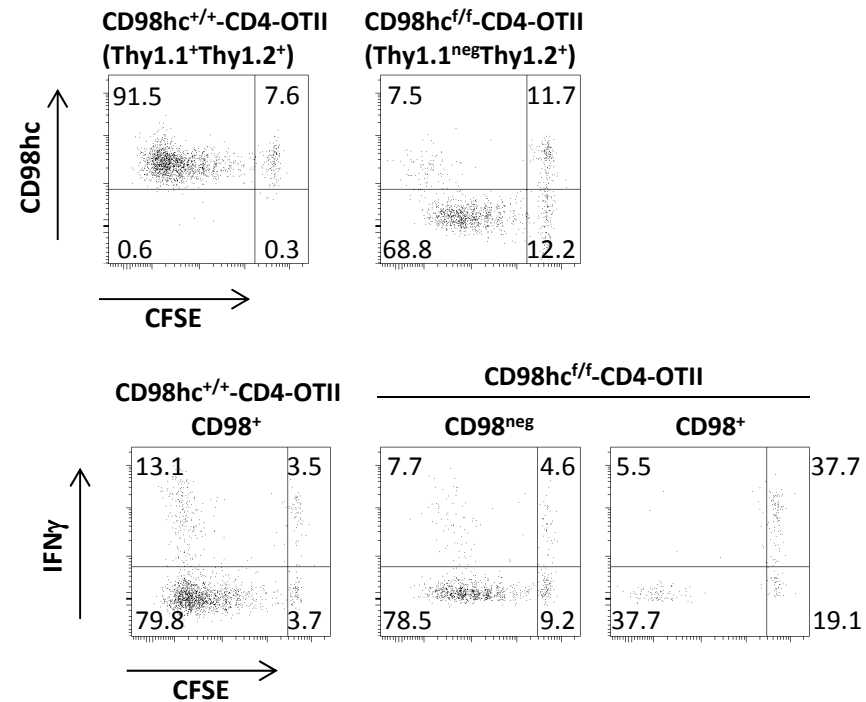

Supplement: S7 Fig — Cells were first analyzed based on their forward scatter and side scatter profiles. Cells were stained with anti-CD45.2, CD45.1, Thy1.2, Thy1.1 and CD4 antibodies and then stained by anti-IFN-γ antibody. (PDF) [file pone.0139692.s007.pdf]
